# June—The Bride Month

**Published:** 1898-06

**Authors:** 


### Selections and Abstracts.

### JUNE—THE BRIDE MONTH.

June, the month of roses, the happy time for mating, the season when

> "So sweet, so sweet the calling of the thrushes,
> The calling, cooing, wooing everywhere."

When breezes blow and deep blue skies are flecked with fleecy clouds. No time of the year has been so favored of the poets, possibly because all Nature seems suggestive of wedded bliss, even the songs of the birds leading to thoughts of happy mating and joyous honeymoons. Perhaps Lowell's lines in his "Vision of Sir Daunfal" breathe the spirit of the month more nearly than any that have ever been written. He says:

> "What is so rare as a day in June?
> Then, if ever, come perfect days;
> Then Heaven tries the earth if it be in tune,
> And over it softly her warm ear lays."

But the physician has little time for looking at the world through poets' eyes. He may have the most delicate aspirations, the most sensitive appreciation of the beautiful, but it is the practical that chiefly claims his attention. As Nature airs her charming garb of green and rose, and breathes the balmy sighs that prefigure the sensuous torrid heats of summer, the doctor is compelled to devote his attention to the seamy side of the picture and resign himself to dancing attendance upon his annual crop of "June colds," early diarrhea, cholera infantum and cholera morbus, with here and there a case of pneumonia. He must take his thoughts from flowers, evening zephyrs, the songs of birds, and consider the contents of a boy's stomach or the condition of a child's bowels.

Early fruits and vegetables, particularly green apples and cucumbers, bring their penalties with their pleasures. The warm days tempt to undue abandonment of clothing, an indiscretion which is promptly avenged by the cool nights; the thirst caused by the increasing temperature is heedlessly gratified by iced drinks and the resulting digestive disorders are apt to be laid to the charge of anything rather than the unhygienic proceedings of the sufferer himself.

There is little doubt but that in the ranks of troubles incidental to this season, disorders of the intestinal tract are *facile princeps*. Consideration, therefore, of the etiology, diagnosis, prognosis and treatment of such is likely to prove of practical value to the general practitioner, and with this point in view *The World* collates what is best in current literature, presenting needful facts in concise form, so that "he who runs may read."

*Acute Diarrhea.*—Possibly the most frequent of the intestinal disorders is acute diarrhea, or catarrhal enteritis. With this trouble there will be a history of injudicious eating, either of unripe fruit or of articles like ice-cream, cream puffs and veal, or even the excessive drinking of milk or water, pure or impure. Tyson says that although "hot weather favors intestinal catarrhs, especially in infants and older children, they are not so much the direct result of the heat as of its effect in weakening the resisting power of the child and favoring the decomposition and fermentation of food. The effect of heat on the nervous system of the very young may reasonably be regarded as a factor in increasing the irritability of the gastro-intestinal tract or so diminishing its functional power as to render the ingesta irritating. Cold, or rather a chilling of the body by a fall in temperature, is often followed by enteritis. Sometimes a hyperactivity of the liver will cause diarrheal discharges."

Anders gives as exciting causes the ingestion of local irritants, overeating, toxic substances, impure water, atmospheric changes, excess or lack of bile. He says these diarrheas have as their chief symptoms slight griping or colicky pains in the abdomen, followed by diarrheal stools, the discharges consisting at first of feculent masses and later of a watery, highly irritating fluid, either bright yellow or yellowish-brown in color, occasionally greenish from the presence of considerable bile pigment or from bacterial action. As a rule there is slight tympanitic distension. Sometimes high temperature is noted, and occasionally great weakness.

The views of the profession concerning the treatment of this trouble have changed greatly. Anders advocates the rigid abstinence from all injurious articles, and, where the cause of the trouble is established to have been the ingestion of such, the giving of a light purgative. Give albuminous food in liquid or semi-liquid form, and enjoin rest in bed. He gives the following prescriptions:

℞ Salol ............................½ dram
Creasoti ............................m x
Bismuthi salicylat.....................1 dram
M. et ft capsulae No. xx. Sig.—
One every three hours.

If pain be troublesome combine opium or phenacetin with the above, or employ:

℞ Argenti nitratis........................gr ii
Ext. opii...............................gr iss
M. et ft. pil No. xii.—Sig. One
every three or four hours.

Other authorities advocate abstention from all food except in the case of infants, the giving of bismuth subnitrate or of chalk mixture and of tannic or gallic acid in five-grain doses, while the various chlorodyns are recommended where there is severe pain. Hot fomentations over the bowels and the enveloping of the abdomen in hot flannel are well spoken of.

*Diarrheas of Children.*—Three forms, more or less distinct, are recognizable in the diarrheas of children. Acute dyspeptic diarrhea, cholera infantum, and acute entero-colitis. The first of these is mainly caused by dietary errors, and is most frequent with "bottle babies." Dentition and excessive heat are also factors in its causation. The attack is initiated with restlessness and slight fever and there is sometimes slight colicky pain, and, rarely, nausea. The stools are at first copious and offensive, often show evidences of fermentation, and often contain particles of undigested food. The trouble may be differentiated from cholera infantum by the lack of serious discharges. The prognosis is often bad among the puny children of the poorer classes. The best remedies available are, first a purge, preferably of calcined magnesia, although castor oil is allowable. This should be followed by bismuth subcarbonate or prepared chalk in doses of 2½ drams with ½ grain doses of salol. Opium should not be given until other means to relieve pain have been exhausted.

A spice plaster applied over the epigastium often proves efficacious. Astringents are seldom indicated. The regulation of the diet is of the utmost importance, and all food that is given should be much diluted. Possibly the best food would be thoroughly boiled milk (not pasteurized), diluted with either lime water or vichy. Give boiled water, afterward cooled to a suitable temperature, but give of both foods and liquids in rather scanty measure. In all infants wrap the body in flannels warmed by dry heat, and see that the room air is kept fresh. If the child is seen and treatment begins before the stage of collapse, advise taking it in the open air carefully wrapped up. Do not sanction too much nursing and holding on the lap, and do not allow natural sleep to be interrupted. The fever that is sometimes observed does not need direct treatment, but will subside with the diarrhea.

When the trouble is once under control care must be taken that matters do not swing too much the other way. It is imperative that the child be kept under observation for some time in order that constipation be not established. It would be well to follow up the general treatment after convalescence by small doses of calcined magnesia given twice a week.

Where it is possible, see that hygienic conditions are established. If the child has been living on some "food," examine into the mother's way of preparing it. If this has been correctly done do not hesitate to order another kind of food, since the first might cause a recurrence of the trouble, adding extraordinarily to the gravity of the prognosis.

*Cholera Infantum.*—Cholera infantum is a variety of acute catarrhal enteritis of intense severity, corresponding in symptoms and course to cholera morbus in the adult, but much more liable to a fatal termination. No specific cause has ever been found for the disorder, but modern writers ascribe it to the irritating effect of toxins generated in the decomposition and fermentation of food, since the history of almost every case discloses some error in diet.

Dentition and weather act as predisposing causes, and it also frequently develops from a mere dyspeptic diarrhea or from an attack of entero-colitis. It is less frequent than some of the other intestinal disorders, Holt claiming that it constitutes not more than two or three per cent. of the summer complaints of children.

The disease is characterized by its copious serous stools, which become ultimately almost like water. They are usually ejected with great force, and this is an important diagnostic point. There is a certain rigidity of the limbs, which may be either drawn up or extended. The fever is decided, sometimes reaching 107°, but averaging 105°. The feebleness and frequency of the pulse are noticeable, and so also is the unusual restlessness.

There is often a cool feeling to the skin, even when the temperature is ranging very high, and for this reason it is well to take the temperature *per rectum*. Occasionally there are intercurrent convulsions, and sometimes all the symptoms attributed to water on the brain are noticed. Sometimes there is torpor and semi-consciousness, with the Cheyne-Stokes respiration.

Intense thirst is always present, and at first the tongue is coated, although it later becomes dry and red. Emaciation is rapid. Simultaneously with the purging is apt to come vomiting, at first greenish from the presence of bile, but later of a serious character. The urine diminishes in quantity, and the abdomen becomes collapsed.

The disease may prove fatal in from one to four days.

The symptoms must be met with the utmost promptness. Opiates are indispensable, according to the best authorities, even morphine hypodermically being permissible if used with great caution in doses of about 1-100 of a grain, associated with 1-500 grain of atropin. Laudanum or the deodorized tincture of opium, in doses of from two to four drops, may be given in starch-water as a rectal injection.

Tyson recommends small doses of Dover's powders, about 1-10 of a grain, in combination with the sub-carbonate of bismuth, in doses of about two grains. He also thinks that preparations of the oxid of silver are sometimes of value, but advocates combining them with opium in the dosage of 1-12 grain of the silver and 1-24 grain of opium.

Leading authorities advocate the use of a mild purgative to empty the intestinal tract, and recommend lavage of the stomach where vomiting is persistent. The colon may also be flushed to advantage.

Among the advocates of enteroclysis, Dr. Thiercelin is prominent. He believes that it is indicated in cases of obstruction of the bowels and in cases in which fermenting materials have been retained in the intestine, whether the disorder be acute or chronic. By this means the fermenting material is removed and the toxins washed out from the bowel. If fever accompanies the infection, cold water may be used, in so large quantity as one or two quarts. A fall of temperature of from 2° to 3° will often follow their use.

Hyperpyrexia is one of the greatest dangers, and this is best combated by hydrotherapy. Ice-bags should be placed to the head, and ice-water injections *per rectum* used. Whenever the temperature rises to an alarming point, the child should be placed in a bath at about 80°, rapidly reduced to 70°. If this cannot be done, the patient should be wrapped in sheets wrung out of cold water. Do not be afraid of being too radical. Sponging is a most inefficacious substitute for these measures.

Since there is so great abstraction of fluid from the system by vomiting and purging, as shown by the intense thirst of the sufferer, it is necessary to supply the losses. There is little use to attempt to give liquid by either mouth or rectum, since it will be rejected by either avenue. Anders advises an injection into the cellular tissues of the buttocks, back or thighs, of a normal saline solution, and says that a pint can be used to advantage every twenty-four hours, while in especially severe cases more may be used.

Drs. M. Barbier and M. Deroyer say that the indications for the use of sterilized normal saline injections are as follows:

1. In acute infection enteritis with low temperature yet without excessive diarrhea.

2. In chronic enteritis which prevents the characteristic of low temperature with progressive weakness.

They do not think the injections are indicated in febrile forms in which it is not necessary to increase the temperature or accelerate the pulse. They are only called for where, during the course of such febrile diarrhea, a period of low temperature or collapse occurs.

In collapse substitute the hot bath for the cold.

Waugh champions the use of the two following prescriptions:

In early stages.

℞ Sodii carb.
Sodii sulfocarbolat........... aa gr xxx
Vini ipecac ...................... dr. j
Tr. castor ...................... dr. ij
Tr. hydrastis..................... dr. iv
Ar. syr. rhei ............ q. s. ad. oz. iv
Sig.—Teaspoonful every two to four hrs.

In the dysenteric stage.

℞ Hydrarg. chlor. mite............ gr. 1/20

Ipecac ......................... gr. 1/20

Testa preparat.................... gr. j

Sig.—Give every two hours until the stools improve.

Bartholow suggests Fowler's solution, 1/8 to 1/4 drop with 1/2 to 1 drop of laudanum, for mucous stools streaked with blood. He also recommends ipecac for greenish stools with blood and mucus, and gives a teaspoonful of the spirit of camphor in four ounces of milk as a stimulant. For diarrhea continuing after the reaction is established, he gives zinc oxid, bismuth and pepsin for the stomach, and copper sulphate for the bowels. If there is nervous irritation or cerebral congestion he prescribes potassium bromid.

Brunton favors carbolic acid and bismuth, and E. Smith pronounces a morphin hypodermic of from one-eightieth to one-thirtieth grain, with five drops of ether, the best treatment.

M. L. Brown has used potassium bromid with great satisfaction in this disease, and considers the drug a specific or physiologic antidote to the toxins that operate as a cause.

Other authorities advocate the use of copper, arsenite, salol, resorcin, and the naphthols.

*Acute Entero-colitis.*—This is an inflammatory process of the ileum and colon, of a greater severity than that of dyspeptic enteritis, and chiefly affects the lymph follicles. It is a disease of the hot months and of teething, although not strictly confined to the former, and is produced by the same causes operative for dyspeptic diarrhea.

Acute entero-colitis has a temperature between that of dyspeptic diarrhea and cholera infantum, and is characterized by the presence of quantities of mucus in the stools, and by absence of vomiting, of colliquative diarrhea, and of collapse.

The prognosis is doubtful, much depending upon the strength of the child and upon whether the surroundings be hygienic.

Treatment should embrace the general dietetic and hygienic precautions advocated for the other forms of intestinal inflammation. Anodynes are necessary. The rectum should be chiefly used for the administration of drugs, particularly for opium when used.

Tyson prescribes solutions of nitrate of silver, one grain to the ounce, or tannic acid, five grains to the ounce, to be given as rectal injections. The mouth should be examined and the gums lanced so often as may be necessary for the coming teeth.

It should be enjoined upon the doctor that he carefully differentiate the form of disease, since the treatment varies in the different cases. Not all cases of infantile summer diarrhea are cholera infantum, and the treatment for cholera infantum would be far from apposite for diarrhea of the acute dyspeptic type. The differential diagnosis is clear and should be closely made.

It is difficult to secure the retention of the stimulants which are so necessary to tide over the period of collapse. Iced champagne may be given in small doses, often repeated, and brandy in cool water will be of benefit. If liquids are rejected, do not be discouraged from repeating their administration.

The matter of the microbal origin of infection in the diarrheal diseases of children has been thoroughly investigated by Dr. Charles G. Cumston, who concludes:

1. The bacterium coli communis appears to be the pathogenic agent in the greater number of cases.
2. This organism is frequently associated with the streptococcus pyogenes.
3. The virulence, more considerable than in the intestine of a healthy child, is almost always in direct relation to the condition of the patient at the time the culture is taken, and does not appear proportional to the ulterior gravity of the case.
4. The motility of the bacterium coli is generally proportional to its virulence. The "jumping" movement, nevertheless, does not correspond to an exalted virulence in comparison with the cases in which the motility was very considerable without exhibiting these jumping movements.
5. The virulence of the bacterium coli found in the blood and other organs is identical to that of the bacterium coli taken from the intestine of the same individual.

*Cholera Morbus.*—This is sometimes called sporadic cholera, and is an acute gastro-intestinal catarrh, characterized by profuse vomiting, purging and painful cramp. The disease is self-limiting, and is characterized by a brief course. The prognosis is generally favorable, although fatal cases sometimes occur in persons suffering from chronic kidney affections or heart disease, or in the very old or very young.

It is supposed to be of microbic origin, although this has not been definitely proved. The "Finkler and Prior" bacillus is found in the discharges with considerable constancy, and this closely resembles the comma bacillus of true cholera. Hence it has by some been considered the exciting cause. Exposure to cold and wet and improper food are often the immediate occasion for the attack.

The onset of the disease is often sudden, beginning with severe vomiting of the last food ingested, then of bile, and lastly of watery fluid. Simultaneously discharges from the bowels will begin, and these will finally assume the "rice water" appearance of true cholera. Following each spasm of vomiting and purging will be severe cramping pains, at first of the abdomen, later extending to the general muscular system, especially to the calves of the legs. This is due to the sudden abstraction of liquids from the system. There is also intense thirst, and generally fever, characterized by a higher internal than external temperature.

Since the symptoms caused by poisoning by arsenic, antimony and toadstools are similar, care must be taken to exclude these. In fact, it has been stated by a high medical authority that three-fourths of the deaths reported as due to cholera morbus are instead cases of arsenical poisoning.

In treatment the diet must be rigorously restricted, predigested foods having the preference when it is necessary to supply nourishment. Opium is almost essential to the successful treatment, but this is best given hypodermically in the form of its alkaloids. Larger doses than usual should be employed, nothing less than at least 1/4 grain being of much use for an adult, and even 1/2 and 5/8 grains being well borne. It is best to begin with the smaller dose and gradually increase until the maximum is reached. Mustard plasters should be applied over the abdomen until the skin shows redness, and then should be followed by linseed poultices to be worn constantly. Absolute rest should be enjoined. If a cold stage supervenes, use the hot bath.

If the immediate cause is the ingestion of irritating substances, it is sometimes well to clear the intestinal tract by a prompt but mild laxative. For the nausea, thirst and weakness, give brandy sprinkled upon chipped ice. If obtainable, the use of champagne will often bring about the happiest results.

If collapse begins, inject whisky and ether under the skin. The injection of large quantities of water into the bowels is advocated to supply the loss of water from the system. If absorption from the bowel is not sufficiently rapid, the normal saline solution may be injected in amounts varying from one-half pint to one quart per day.

Waugh advocates the administration of five drops of chloroform in a little water every five minutes until the pain is controlled, and says that hot applications to the epigastrium or over the right pneumogastric nerve in the neck are valuable adjuvants. He gives this prescription:

℞ Camphoræ
Chloroformi....................aa. dr. ij
Ol. cajeput........................dr. iv
Etheris
Tr. capsici.......................aa. oz. j
M. Sig.—Teaspoonful in very little water every 15 minutes until reaction occurs.

For after treatment he gives:

℞ Bismuthi subnitrat .............. dr. ij
Sodæ sulfocarbol ...............gr. xviij
Ar. syr. rhei.......................oz. vj
Sig.—Tablespoonful every two hours.

A woolen bandage should be worn after recovery and the after-diet should be particularly bland and unirritating.

Ringer prescribes camphor, gives veratrum album for vomiting and in the earlier stages gives lead acetate.

Da Costa prescribes the usual eliminative and anodyne treatment with the addition of chloral, one part, to five of soap liniment, rubbed on the abdomen for cramps.

Bartholow claims that chloral by enema is the best treatment, but says that carbolic acid with bismuth is very efficient. He gives morphine hypodermically in very full doses and iced brandy for the vomiting.

Other remedies recommended are: Ipecac by Waring (who does not believe in the use of stimulants), and columbo as an anti-emetic by Phillips, who also thinks sumbul and oil of cajeput excellent remedies. For heart failure Phillips gives a mustard emetic.

*Hay Fever, "June Cold," or "Rose Cold."*—This is a catarrhal inflammation of the upper air-passages, occurring through the summer months, and is associated with asthmatic dyspnea. It is most intractable to medical treatment, and seems little benefited by anything short of change of scene.

Occasionally operative treatment on the nasal cavities, such as correcting deviations, removal of hypertrophic processes by the knife, or actual cautery, have produced total cures. Palliative treatment is most uncertain.

Irrigating the nasal passages with a weak quinin solution has sometimes proved very beneficial. Internally quinin and Fowler's solution seem to give the most beneficial results; but the administration of chloral in small doses (2½ grains), frequently repeated, has many advocates.

Anders has had the happiest results from the use of atropin in small doses, either hypodermically or by the mouth. He gives about the one-tenth of a grain every hour or one three-hundredth of a grain every half-hour during the first few days. He thinks the attack is controlled better by this small dosage than by larger amounts given less frequently.

The above covers the field of the diseases especially likely to be encountered during June, and is a summary of the best thoughts of the leading authors in this country and abroad. Still, it is possible that sometimes cases may occur which are not typical in their course, and yet require immediate attention and the promptest treatment. In such event it is almost always safe to assume that cases of intestinal inflammation are due to the presence of irritating substances, and whether such are merely articles of food or are some specific microbe, it is well to adopt an antiseptic mode of treatment for the general indications, and to prescribe anodynes for the attendant pain. Stimulants are always necessary for symptoms of collapse.—*Medical World.*

